# Disrupted Functional Connectivity with Dopaminergic Midbrain in Cocaine Abusers

**DOI:** 10.1371/journal.pone.0010815

**Published:** 2010-05-25

**Authors:** Dardo Tomasi, Nora D. Volkow, Ruiliang Wang, Jean H. Carrillo, Thomas Maloney, Nelly Alia-Klein, Patricia A. Woicik, Frank Telang, Rita Z. Goldstein

**Affiliations:** 1 National Institute on Alcohol Abuse and Alcoholism, National Institutes of Health, Bethesda, Maryland, United States of America; 2 National Institute on Drug Abuse, National Institutes of Health, Bethesda, Maryland, United States of America; 3 Medical Department, Brookhaven National Laboratory, Upton, New York, United States of America; 4 Computer Science Department, SUNY at Stony Brook, Stony Brook, New York, United States of America; Cuban Neuroscience Center, Cuba

## Abstract

**Background:**

Chronic cocaine use is associated with disrupted dopaminergic neurotransmission but how this disruption affects overall brain function (other than reward/motivation) is yet to be fully investigated. Here we test the hypothesis that cocaine addicted subjects will have disrupted functional connectivity between the midbrain (where dopamine neurons are located) and cortical and subcortical brain regions during the performance of a sustained attention task.

**Methodology/Principal Findings:**

We measured brain activation and functional connectivity with fMRI in 20 cocaine abusers and 20 matched controls. When compared to controls, cocaine abusers had lower positive functional connectivity of midbrain with thalamus, cerebellum, and rostral cingulate, and this was associated with decreased activation in thalamus and cerebellum and enhanced deactivation in rostral cingulate.

**Conclusions/Significance:**

These findings suggest that decreased functional connectivity of the midbrain interferes with the activation and deactivation signals associated with sustained attention in cocaine addicts.

## Introduction

With repeated use, cocaine leads to neuroadaptations in dopaminergic function (as well as neuroadaptations in other catecholamines and glutamatergic and gabaergic systems) [1]–[4]. These neuroadaptations could interfere with the functional connectivity of brain regions modulated by dopamine and thus contribute to the decreased reward sensitivity, enhanced stress reactivity, and executive cognitive dysfunction reported in cocaine abusers [1], [5]–[9].

The fluctuations of neural activity that mediate neuroadaptations [10] can alter dynamically the cerebral blood flow and volume [11] and produce synchronous magnetic resonance imaging (MRI) signals in different brain regions [12]. This synchronous MRI signal fluctuations have been used to assess the in-vivo functional connectivity of the human brain in resting-state conditions [13]. Here we studied the correlation between signals in midbrain, which is where dopamine (DA) neurons are located, and those in cortical and subcortical structures during sustained attention conditions as a way to assess the modulatory strength of the dopaminergic pathway on cognition in cocaine addiction. We used a sustained attention drug-word (DW) task that tests the processing of drug vs. matched neutral words [14]. Using this DW task we showed that compared to controls cocaine addicts had higher drug-cue related activation in midbrain [15], and hypo-activation in anterior cingulate cortex [16]. Based on our previous findings [2], [15]–[18] and preclinical studies documenting disruption of dopaminergic pathways with repeated cocaine administration [19], [20], we hypothesized that hypoactive brain regions in cocaine abusers would also show lower functional connectivity with midbrain.

## Methods

### Subjects

Twenty healthy chronic cocaine abusers and 20 age-, gender-, and education-matched healthy control subjects participated in this study (Table 1). Participants were recruited from advertisements on public bulletin boards, in local newspapers, and by word-of-mouth. Eligible subjects were scheduled for an onsite evaluation that included a full physical and neurological examination by a neurologist. All subjects provided written informed consent as approved by the local Institutional Review Board (Stony Brook University's Committee on Research Involving Human Subjects, CORIHS), and were screened for absence of medical, psychiatric or neurological diseases. A clinical psychologist conducted a semi-structured diagnostic interview which included the Structured Clinical Interview for DSM-IV Axis I Disorders [research version [21], [22]] and the Addiction Severity Index [23].

**Table 1:** Subject characteristics.

Category	Cocaine (N = 20)	Control (N = 20)
Number of men/women	17/3	13/7
Ethnicity Caucasian/Asian/Hispanic/African-American	1/0/2/17	5/1/3/11
Age (years)	41.4±5.0	38.3±10.3
Education (years)	13.1±1.3	14.1±3.2
Verbal IQ	98.8±9.4	102.4±21.3
Cigarette smoking (current or pass/never)	15/5	5/15
DSM-IV diagnoses of cocaine dependence/abuse	16/3	−
DSM-IV diagnoses of cannabis/alcohol abuse	1/1	−
Positive cocaine toxicology screen	17	−
Age at onset of cocaine use (years)	23.9±5.7	−
Cocaine duration (years)	15.3±4.9	−
Frequency of cocaine use (days/week)	3.9±2.2	−

Subjects were included in the study if they were (1) able to understand and give informed consent; and (2) had at least 12 years of education (or equivalent) and verbal intelligence ≥85 (estimated with the Wide Range Achievement Test reading subtest standard score). Subjects were excluded if they had: (3) history of head trauma or loss of consciousness (greater than 30 minutes) or other neurological disease of central origin (including seizures); (4) abnormal vital signs at time of screening or history of major medical conditions that may alter cerebral function, such as cardiovascular (including high blood pressure), endocrinological (including metabolic), oncological or autoimmune diseases for which the participant is required to take medications; (5) present or past history of psychiatric disorders other than substance abuse in cocaine abusers and nicotine dependence in all subjects; (6) positive urine screens for psychoactive drugs (or metabolites) other than cocaine (phencyclidine, benzodiazepines, cannabis, opiates, barbiturates and inhalants); (7) pregnancy, as confirmed with a urine test in all female subjects of childbearing age on study day; and (8) metal implants or other contraindications for MRI.

Differences in age, education, and verbal IQ between the groups were not statistically significant (p>0.07, two-sample t-test). Similarly, the gender difference between groups was not statistically significant (*p* = 0.27; Fisher's exact test). There were also no significant differences in symptoms of depression, as assessed with the Beck Depression Inventory II edition [24], between the cocaine and control groups (p = 0.093; two-sample t-test). Fifteen cocaine subjects were cigarette smokers while only five control subjects were cigarette smokers; the difference in cigarette smoking between the groups was statistically significant (*p* = 0.004; Fisher's exact test). Subjects were allowed to smoke regularly to minimize withdrawal symptoms. Specifically, seven cocaine subjects smoked within 4 hours prior the study and 4 additional cocaine subjects smoked within 6 hours prior the study. There was no difference in time since last cigarette (within 4 hours, >4 hrs, overnight or more) or in smoking frequency between the groups (P>0.54 t-test). Nineteen cocaine subjects met DSM-IV diagnosis for cocaine dependence or abuse, and had at least a 12-month (4 days/week for all but one subject) history of cocaine use (predominantly by the smoked route). Two cocaine dependent subjects also met diagnosis for cannabis or alcohol abuse. Two subjects had histories of past alcohol dependence. Seventeen of the cocaine subjects had a positive urine toxicology screen for cocaine on the day of the study, indicating that they used cocaine during the prior 72 hours. The urine toxicology screen was negative for the remaining three cocaine subjects and for all control subjects.

### Stimulation paradigm

**Figure 1:**
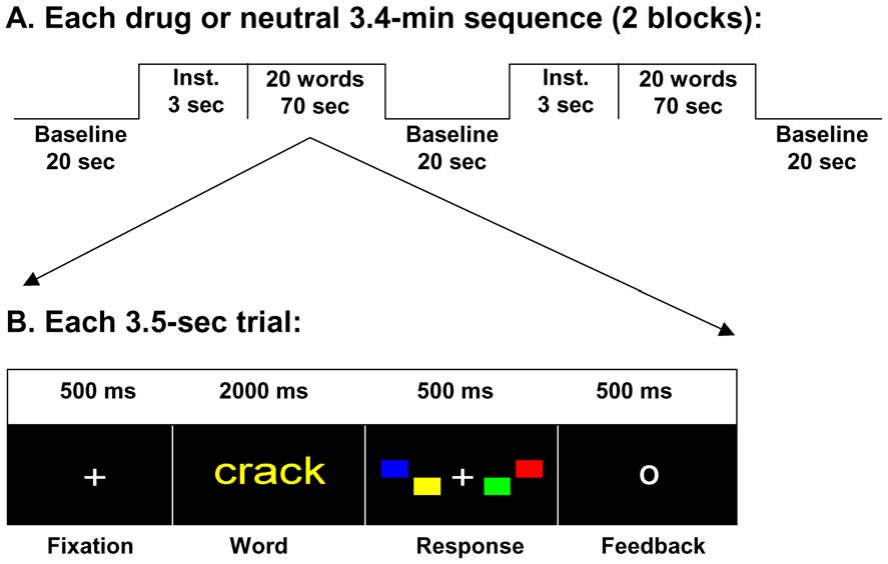
Drug-word fMRI paradigm.

We used a drug-word (DW) paradigm that has been previously described [14]. Briefly, 40 drug words matched on length, frequency in the English language, and part of speech (noun, adjective, adverb, verb) with 40 household-related (neutral) items were used (Fig. 1); non-English words or slang words that may have not been recognized by the control subjects were not included. These two word types (“Drug” and “Neutral”) were presented via MRI-compatible goggles in a blocked and counterbalanced fashion across subjects. Each of the 2 DW sequences (“drug” or “neutral”) had two 70-seconds long task epochs that included 20 trials. Each trial comprised a 500 ms fixation cross, a 2000 ms word presentation window, a 500 ms response window, and a 500 ms feedback where “O” and “X” were displayed for correct and incorrect trials, respectively. The words were presented using one of four possible colors (yellow, blue, red, green) and the word color order was pseudo randomized. Subjects were instructed to press one of four buttons (yellow, blue, red, green) of the MRI-compatible Lumina LP-400 response pad (Cedrus Corp., San Pedro, CA) during the response window matching the color of the word. A fixation cross against a black background was presented during the three 20-seconds long resting baseline epochs and subjects were instructed to not press any button and rest motionless as possible during these epochs. Thus, each sequence (“drug” or “neutral”) was 206 seconds long. The task was developed in E-prime (Psychology Software Tools, Inc., Pittsburgh, PA) and used a trigger pulse from the MRI console for precise synchronization with fMRI acquisition.

### Data acquisition

A 4-Tesla whole-body Varian/Siemens MRI scanner and a T2*-weighted single-shot gradient-echo planar imaging (EPI) pulse sequence (TE/TR = 20/1600 ms, 4-mm slice thickness, 33 coronal slices, 64×64 matrix size, 3.1×3.1 mm in-plane resolution, 90°-flip angle, 131 time points) were used to map the blood oxygenation level dependent (BOLD) fMRI responses in the whole brain. Padding was used to minimize motion. Subject motion was monitored immediately after each fMRI run using a k-space motion detection algorithm [25] written in IDL (ITT Visual Information Solutions, Boulder, CO). Earplugs (acoustic noise attenuation: 28 dB; Aearo Ear TaperFit 2; Aearo Company) and headphones (acoustic noise attenuation: 30 dB; Commander XG MRI Audio System, Resonance Technology inc.) were used to minimize the interference effect of scanner noise during fMRI [26].

Anatomical images were collected using a T1-weighted 3D-MDEFT sequence [27] (TE/TR = 7/15 ms, 0.94×0.94×1.00 mm^3^ spatial resolution, axial orientation, 256 readout and 192×96 phase-encoding steps, 16 minutes scan time) and a modified T2-weigthed Hyperecho sequence [28] (TE/TR = 42/10000 ms, echo train length  = 16, 256×256 matrix size, 30 coronal slices, 0.86×0.86 mm^2^ in-plane resolution, 5-mm thickness, no gap, 2 min scan time), and reviewed to rule out gross brain morphological abnormalities.

### BOLD-fMRI analyses

Image reconstruction was performed using an iterative phase correction method in IDL that minimizes signal-loss artifacts in EPI [29]. The first four volumes in the time series were discarded to avoid non-equilibrium effects in the fMRI signal. Subsequent analyses were performed with the statistical parametric mapping package SPM2 (Welcome Department of Cognitive Neurology, London UK). The images were motion corrected with a 12-parameter affine transformation, spatially normalized to the standard brain (using a 12-parameters affine transformation with medium regularization and 16-nonlinear iterations and voxel size of 3×3×3 mm^3^), and smoothed (8-mm full-width-half-maximum Gaussian kernel). Note that spatial normalization to the stereotactic space was carried out using the standard SPM2 EPI template.

The general linear model used to calculate the individual BOLD contrast maps (drug epochs and neutral epochs) for each fMRI time series consisted of a box-car design convolved with the canonical hemodynamic response function (HRF) and a high-pass filter (cut-off frequency: 1/520 Hz). The BOLD signal strength was estimated without the removal of global effects (global normalization) to minimize false deactivation signals [30], [31]. The estimated BOLD maps, contrasting word epochs (drug or neutral) against baseline epochs, were included in a two-way (word type × group) repeated measures analysis of variance (ANOVA) model in SPM2. Four covariates (smoking status, urine results, gender, and age) were included in this random-effects model in an effort to control for potential confounds. Brain activation clusters were corrected for multiple comparisons using the continuous random field calculation implemented in SPM2. Clusters with at least 15 voxels (400 mm^3^) and *p_corr_* <0.05, corrected for multiple comparisons at the cluster level, were considered significant in group analyses of brain activation.

### Functional Connectivity

**Figure 2:**
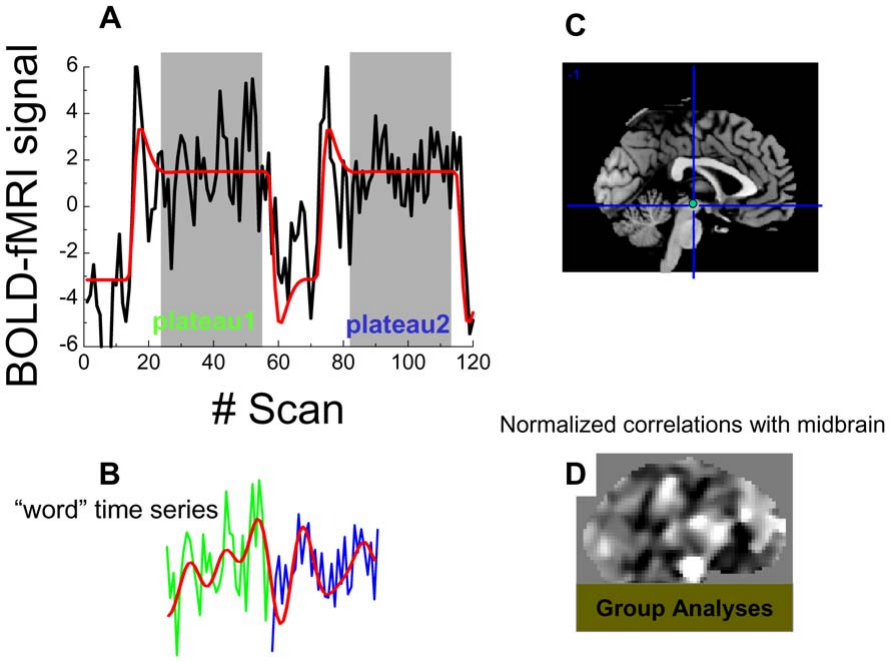
Functional connectivity analysis of task-related signal fluctuations during the blocked fMRI paradigm. **A**: BOLD response elicited by the drug-word paradigm (black curve) and the fitted SPM2 canonic hemodynamic response (red curve). The gray periods identify the plateaus of the model. **B**: “Word” time series are composed by 37 the time points of plateau 1 (green curve) and the 37 time points of plateau 2 (blue curve). **C**: Mid-sagittal slice of an MRI structure showing the position of the midbrain seed used for the functional connectivity analysis. D: Mid-saggital slice exemplifying the normalized (z-score) maps reflecting correlations of MRI signals in the brain with those in midbrain. These individual maps were used in group analyses of functional connectivity.

A method recently proposed for studies based on blocked fMRI datasets [32] was used to evaluate the functional connectivity of the brain using the resting epochs. Here, we were interested in the functional connectivity of the midbrain during the performance of the sustained attention condition of the drug-word task, not during resting-conditions. The task design was advantageous for this purpose because the “word” epochs were 70 seconds long, almost double the length of the resting blocks used by Fair and colleagues. In order to minimize unwanted task-related effects on the strength of correlations with midbrain (CM) we discarded 11 seconds from each word epoch, using the canonic hemodynamic response function to optimally select time points of the word epochs. Specifically, for each time series, the plateaus of the SPM2 canonic hemodynamic response function (Fig. 2A, red curve) were used to identify the 37 consecutive time points of the plateaus (Fig. 2B green and blue curves) corresponding to “word” epochs. This procedure accounted for the initial delay of the hemodynamic response and its return to baseline. The selected time points corresponding to each word epoch were concatenated to form “word” (“Drug” or “Neutral”) time series with 74 time points. “Word” time series were carefully inspected to ensure minimal baseline differences at the concatenation time points, and band-pass filtered (0.01–0.1 Hz; Fig. 2B, red curve), a step that further minimizes this baseline differences. A midbrain region (left substantia nigra, SN; talairach position: *xyz* = [−6, −15, −18] mm; Fig. 2C, green circle) that showed higher drug-cue related activation for cocaine addicts than for controls using a more complex version of the DW task [15] was selected as the “seed” voxel for the functional connectivity analysis. Whole-brain maps reflecting correlations between BOLD signals in the seed and those in all other voxels in the brain were calculated separately for each “word” time series. The Fisher transform was used to convert the step distributed Pearson linear correlation factors into normally distributed CM coefficients. These normalized CM maps (Fig. 2D) were computed and saved in Analyze format using IDL, and loaded into SPM2 for group analyses of CM [33]. A two-way (word type × group) repeated measures ANOVA (random-effects) model with four covariates (smoking status, urine results, gender, and age) was used for group analyses of CM. Clusters with at least 15 voxels and *p_corr_* <0.05 (corrected for multiple comparisons) were considered significant in group analysis of CM signals.

### Regions-of-interest (ROI) analyses

The relevant clusters were further evaluated with region-of-interest (ROI) analyses to identify potential outliers that might influence statistical analyses, and to report average values in a volume comparable to the image smoothness (e.g. resolution elements, or “resels” [34]) rather than single-voxel peak values. The volume of the resels was estimated using the random field calculation in SPM2 as a near cubic volume with Cartesian full-width-half-maximum (FWHM) of [13.0, 12.3, 13.4] mm for group analyses of brain activation and of [11.8, 11.0, 12.4] mm for group analyses of CM. Note that (2n+1)^3^ voxels fit into ROI that are symmetric around a peak voxel and that 3-mm isotropic voxels were used. We did not use n  = 0 ROIs because do not take full advantage of the 13-mm isotropic resels (include only one voxel). Similarly, n = 2 ROIs were not used because they include voxels that do not belong to the average functional cluster defined by the resels. Thus, n = 1 ROIs were used to maximize the number of voxels within the smoothness of the data. Thus, the average BOLD and CM signals in the left and right medial dorsal nuclei of the thalamus (MDTHA; xyz = ±3, −15, 9 mm), cerebellum (CER; culmen; xyz = ±3, −45, −6 mm), and the rostral anterior cingulate cortex (rACC; xyz = ±3, 42, −3 mm) were extracted from the individual BOLD and CM maps in 9×9×9 mm^3^ ROI volumes containing 27 voxels to using IDL. We selected these ROIs based on our previous fMRI studies [16]–[18], [35]. These ROI masks were created and centered at the precise coordinates listed in Table 2; the coordinates of the ROI masks were kept fixed across subjects and conditions. Note that compared to variable-volume ROIs, fixed-volume (shape and size) ROIs are advantageous in order to minimize statistical confounds. Brain regions were labeled using the Talairach daemon (http://www.talairach.org/) [36] and a query range of 5 mm.

**Table 2:** Statistical significance (t-score) of brain activation during the DW paradigm for cocaine and control subjects and their location in the Talairach frame of reference (x, y, z).

Region	BA/nucleus	Cocaine (t)	Control (t)	X [mm]	Y [mm]	Z [mm]
Fusiform Gyrus	19	17.1	16.4	24	−81	−12
Lingual Gyrus	18	23.9	25.0	−24	−78	−9
Medial Frontal Gyrus	6	12.8	16.0	3	3	54
Middle Frontal Gyrus	6	4.2	2.7	−27	0	48
Superior Frontal Gyrus	6	7.2	9.2	3	−3	66
Precentral Gyrus	6	NS	2.7	45	−3	36
Insula	13	5.1	7.1	36	12	9
Insula	13	5.7	6.9	−45	9	6
Precuneus	7	5.9	6.1	30	−45	48
Inferior Frontal Gyrus	10	3.6	2.5	−36	45	3
Thalamus*	Medial Dorsal	3.9	8.0	−3	−15	9
Thalamus*	Medial Dorsal	4.4	8.9	3	−15	9
Cerebellum*	Culmen	NS	5.5	−3	−45	−6
Cerebellum*	Culmen	3.6	7.7	3	−45	−6
Anterior Cingulate*	32	−13.3	−4.7	−3	42	−3
Anterior Cingulate*	32	−8.9	NS	3	42	−3
Cuneus	18	−5.1	−6.0	−15	−78	24
Precuneus	7	−6.5	−6.6	−12	−48	39
Precuneus	19	−3.9	−7.8	36	−72	39
Middle Temporal Gyrus	39	−4.6	−8.8	42	−72	24
Sub-Gyral	21	−2.1	−2.7	−39	−12	−9
Parahippocampal Gyrus	36	−3.5	−4.7	24	−42	−6
Parahippocampal Gyrus	28	−2.9	−3.4	−18	−21	−18
Insula	13	NS	−2.7	−39	−12	18
Insula	13	−4.6	−4.1	36	−18	18

Cluster statistics: P_corr_ <0.0005, familywise error corrected for multiple comparisons using the random field theory.

*indicates clusters showing statistically significant between-group differences P_corr_ <0.05.

## Results

### Performance

**Figure 3:**
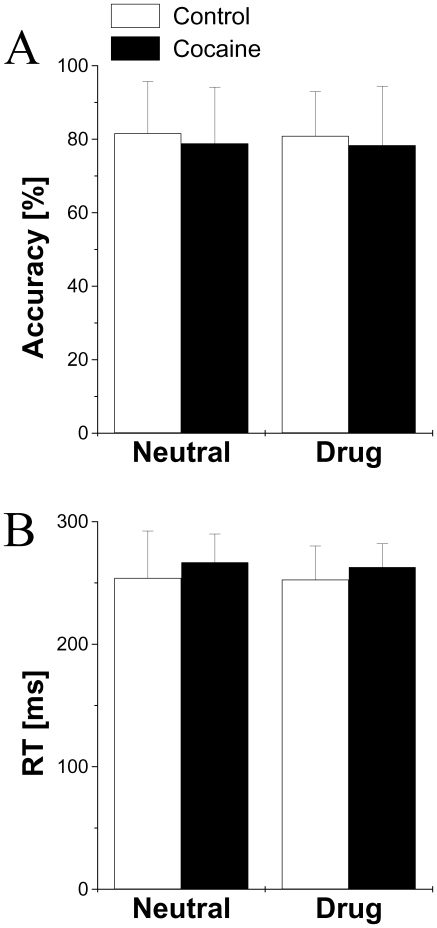
Behavioral data. Average performance accuracy (A) and reaction times (RT; B) during the drug-word (DW) paradigm for cocaine (N = 20) and control (N = 20) subjects.

Differences in performance (accuracy and reaction time, RT) during the DW task were not statistically significant, either between groups (cocaine subjects vs. controls; accuracy: p = 0.55, F = 0.36, DF = 1; RT: p = 0.18, F = 1.89, DF = 1; repeated measures ANOVA) or between words (“Neutral” vs. “Drug”; accuracy: p = 0.64, F = 0.22, DF = 1; RT: p = 0.39, F = 0.77, DF = 1, Fig. 3). Similarly, group × word interaction effects on behavioral responses were not statistically significant (accuracy: p = 0.93, F = 0.009, DF = 1; RT: p = 0.66, F = 0.19, DF = 1).

### Brain activation

**Figure 4:**
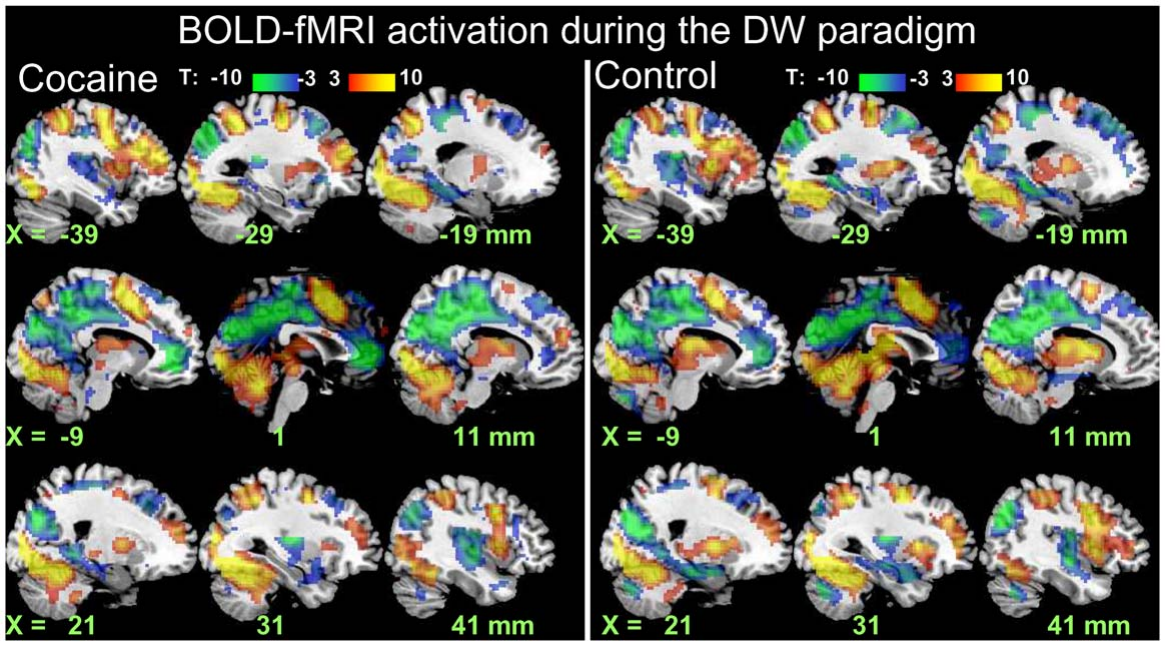
Brain activation. Statistical maps of BOLD-fMRI signals during the DW task across word conditions (“Drug” and “Neutral”) for 20 cocaine abusers (left) and 20 healthy matched control subjects (right). Random-effects analyses (two-way repeated measures ANOVA). Red-yellow and blue-green color bars show the *t*-score windows for activation and deactivation, respectively.

**Figure 5:**
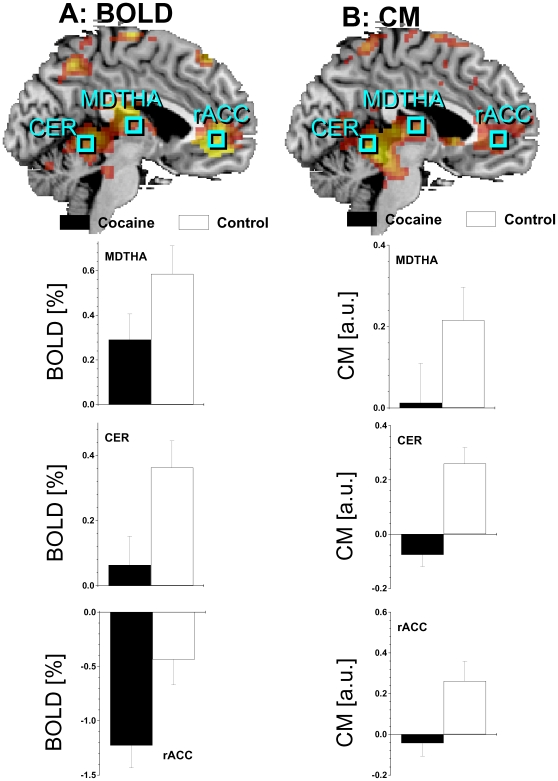
Brain activation and functional connectivity of midbrain. A: Statistical map of BOLD-fMRI signals during the DW task across word conditions (conjunctive analysis “Drug” + “Neutral” vs. resting baseline) for controls > cocaine, superimposed on a sagittal view of the human brain. The light-blue squares and labels mark the positions of relevant ROI. The left side bar plots display the average BOLD responses in these regions for the cocaine and control groups (P<0.05). 5B: Statistical map of correlations with midbrain (CM) across word conditions (conjunctive analysis “Drug” + “Neutral” vs. resting baseline) for controls > cocaine, superimposed on a sagittal view of the human brain. The right side bar plots display the average CM signals in these regions for the cocaine and control groups (P<0.05). SPM Model: two-way repeated measures ANOVA. Sample: cocaine (N = 20) and control (N = 20) subjects. MDTHA: medial dorsal nucleus of the thalamus; CER: cerebellum (culmen); rACC: rostral anterior cingulate cortex (BA 32). ROI volume = 27 voxels (0.73 cc). Error bars are standard errors. CER: cerebellum; MDTHA: medial dorsal nucleus of the thalamus; rACC: rostral Anterior cingulate cortex.

In both groups, the DW paradigm activated a bilateral network that included the prefrontal cortex (PFC) [caudal dorsal anterior cingulate Brodmann area (BA) 32, inferior (BA 47), and middle (MFG; BA 9) frontal gyri], inferior (BA 40) and superior (BA 7) parietal cortices, thalamus, dorsal striatum, midbrain, and cerebellum and deactivated the parahippocampal (BA 30) and rostral anterior cingulate (rACC, BA 32) gyri, precuneus (BA 31), amygdala, and insula (BA 13) (*p*_corr_ <0.001, cluster-level corrected for multiple comparisons; Fig. 4 and Table 2). Group comparisons showed that activation was higher for controls than for cocaine abusers in the dorsolateral PFC (MFG BA 9, and precentral gyrus BA 6), cerebellum, thalamus, and left caudate (*p*_corr_ <0.05; Fig. 5A and Table 2); cocaine abusers deactivated more the rACC than controls (*p*_corr_ <0.05; Fig. 5A and Table 2). There were no statistically significant word-related differences (“Drug” > “Neutral” or “Neutral” > “Drug”) on brain activation. There were no statistically significant word × group interaction effects on brain activation.

### Functional Connectivity

**Figure 6:**
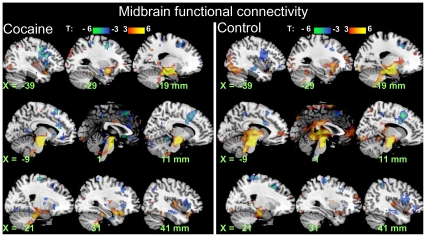
Functional connectivity of midbrain. Statistical maps of normalized CM coefficients during task epochs of the DW task across word conditions (“Drug” and “Neutral”) for 20 cocaine abusers (left) and 20 healthy matched control subjects (right). Random-effects analyses (two-way repeated measures ANOVA). Red-yellow and blue-green color bars show the *t*-score windows.

Across subjects, bilateral occipital (BAs 17-19 and 37), temporal (BA 21), and cingulate (BAs 24, 30) cortex, ventral precuneus (BA 7), thalamus, parahippocampus, amygdala, putamen, caudate, cerebellum, and pons had positive CM (*p*_corr_ <0.001; Fig. 6); temporal (BAs 20, 22), parietal (BAs 2, 3, and 5), prefrontal (BA 9), cingulate (BA 32), and orbitofrontal (BAs 11 and 47) cortex, and posterior insula had negative CM (*p*_corr_ <0.001; Fig. 6). The CM in MDTHA, cerebellum and rACC was higher for controls than for cocaine abusers (*p*_corr_ <0.05; Fig. 5B). There were no statistically significant word-related differences in whole-brain analyses of CM.

### ROI results

**Figure 7:**
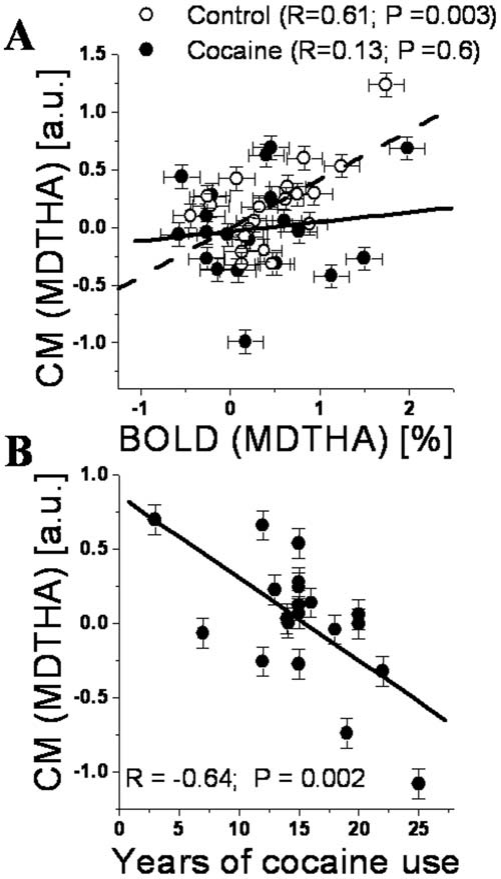
Midbrain-thalamus functional connectivity of vs. thalamic activation and cocaine exposure. Scatter plots of average CM coefficients vs. the average BOLD responses (A) and the duration of cocaine use in the life span (B) for the dorsal medial nucleus of the thalamus. Solid circles: cocaine addicts; open circles: controls. Solid and dashed lines are the corresponding linear fits. ROI volume = 27 voxels (0.73 cc). Error bars are standard errors. MDTHA: medial dorsal nucleus of the thalamus.

CM and BOLD signals in the thalamus were significantly correlated for control subjects (R = 0.61; P = 0.003; Fig. 7A) but not for cocaine subjects. CM signals in the thalamus were correlated negatively with the number of years of cocaine use in the cocaine subjects (R = −0.64; P = 0.002; Fig. 7B). BOLD and CM signals in CER and MDTHA did not show statistically significant correlations with self-reported time since last use of cocaine. However, CM (but not BOLD) signals in rACC were significantly correlated with self-reported time since last use of cocaine (P_c_ = 0.008, Bonferroni corrected for multiple comparisons; Z = 2.8).

## Discussion

Here we show that the midbrain's positive functional connectivity with MDTHA, cerebellum (culmen), and rACC was significantly lower for cocaine subjects than for controls. In parallel and in absence of performance differences, cocaine subjects had lower activation in MDTHA and cerebellum and higher deactivation in rACC than controls, which is consistent with our previous findings in cocaine subjects tested with working memory [18] and visual attention tasks [17].

Using the DW task we recently showed that drug words, but not neutral words, activated the midbrain in cocaine subjects but not in controls [15]. In the current work we evaluated the functional connectivity of this midbrain region (Talairach coordinates *xyz* = [−6, −15, −18] mm) with the rest of the brain using a different sample and a simplified version of the stimulation paradigm. Since the midbrain is relatively small and the imaging smoothing was larger than 12-mm is all directions, the functional responses in the selected seed region are representative of those in the entire midbrain. The midbrain (mesencephalon) is the origin of the main dopamine (DA) projections to the forebrain, and cocaine addicts have lower dopaminergic function than controls [5]. Since cocaine binds to norepinephrine (NE) and DA transporters [37], thereby increasing extracellular NE and DA [1], [38], [39], chronic cocaine exposure could be additionally associated with disrupted neurotransmission in noradrenergic pathways including those into the thalamus. Indeed recent imaging studies have provided evidence of noradrenergic abnormalities in the thalamus (including medial dorsal nuclei) of cocaine abusers when compared with controls [40]. Because midbrain also includes the upper portion of the locus coeruleus, the main norepinephrinergic nucleus in the brain, and the upper part of the rostral raphe, which is the main source of serotonergic innervation to cerebellum and forebrain, and considering that the spatial resolution of fMRI is limited to few mm [41], altered functional connectivity of the midbrain with MDTHA, cerebellum, and rACC could reflect not just neuroadaptations in dopaminergic but also in norepinephrinergic and/or serotonergic neurotransmission with chronic cocaine exposure.

The MDTHA had lower CM and lower BOLD signals for cocaine abusers than for control subjects. These results are consistent with previous studies that documented reduced DA release [5] and reduced activation [17], [18], [42] in the MDTHA for cocaine abusers compared to control subjects. The MDTHA is innervated by norepinephrinergic and dopaminergic neurons [43]. Our current findings may therefore reflect a dysfunctional norepinephrinergic and dopaminergic regulation of the MDTHA in cocaine addicts. This interpretation is further supported by the negative correlation between the thalamic CM signals and the years of cocaine use, which suggests that functional connectivity could be a better marker than BOLD responses for the precise characterization of drug-related neuroadaptations. The positive correlation of thalamic BOLD and CM signals in control subjects and the lack of similar correlations in cocaine subjects further supports this conclusion.

This study also shows an association between hypo-activation and lower CM in the cerebellum for cocaine subjects compared to controls. The locus coeruleus, a homeostatic gray matter nucleus in the brainstem (lower midbrain/pons), is the main source of norepinephrinergic innervation to the thalamus and cerebellum [44] and is closely located to our midbrain seed (22 mm; 7 imaging voxels apart from midbrain; Fig. 7). These results are therefore consistent with altered norepinephrinergic regulation in cocaine addiction. Since most cocaine subjects had a positive urine for cocaine on the day of the study, the cocaine subjects' lower cerebellar BOLD and CM signals may reflect in part activation of norepinephrinergic pathways during short-term/acute withdrawal [45]. However, it could also reflect serotonergic dysfunction since there is increasing evidence of serotonergic regulation of cerebellar activity [46].

The negative BOLD responses in the rACC were higher and the CM signals in rACC were lower for cocaine abusers than for control subjects. The rACC, which is innervated by DA, noradrenergic [47] and serotonergic [48] neurons has been implicated in generation or regulation of spontaneous internal thoughts, emotions, anxiety and in internal conflict resolution [49]–[52]. Indeed, the rACC activates during emotional tasks and deactivates during cognitive tasks [53], [54]. Taking into account that compared to controls cocaine subjects showed rACC hypoactivation during this [16] and other [17], [18], [55] fMRI paradigms, the higher negative BOLD responses and the lower CM signals in rACC may reflect lower DA-mediated suppression of task-irrelevant emotional responses during fMRI in cocaine abusers.

### Study limitations

We did not exclude smokers as from our experience around 75% of cocaine subjects smoke cigarettes (vs. around 25% of controls). Fifteen cocaine subjects and five control subjects were smokers, and the group difference was statistically significant. Subjects were allowed to smoke regularly to minimize withdrawal symptoms. Previous studies have shown that nicotine can induce a dose-dependent increase in neuronal activity in a distributed system of brain regions, including the nucleus accumbens, amygdala, cingulate, and frontal lobes [56]. Therefore, the blunted responses of the cocaine smokers could have been even more suppressed if they would have been abstinent for nicotine prior the study. Taking into account that the average elimination half-life for plasma nicotine is 2 hours in humans [57], and given that none of the controls subjects and only two cocaine subjects smoked within the 2-hours period before the study, none of them smoked during the study, and there was no difference between groups in time since last cigarette, the potentially circulating nicotine levels should have been low and the differential effects of nicotine on brain activation should be minimal. Note that contrasting with our previous study [15], we did not observe drug vs. neutral word activation differences in midbrain in the present study, probably reflecting the lack of monetary incentive conditions and the lower statistical power associated to the simplified version of the DW paradigm.

Summary: Using high-field (4 Tesla) fMRI, here we show that during processing of drug and matched neutral words cocaine abusers have similar accuracy and reaction times to matched controls, but lower BOLD and CM signals in the dorsal medial nucleus of the thalamus, cerebellum, and rostral anterior cingulate cortex. These findings suggest that lower recruitment of subcortical resources and impaired inhibition of cortical resources may be mediated by abnormal functional connectivity of catecholamine (dopamine, norepinephrine and serotonin) pathways in cocaine abusers. The negative correlation of the thalamic CM (and the lack of correlation with BOLD responses in the thalamus) with years of cocaine use suggests that functional connectivity might be more sensitive than standard fMRI activation techniques for the detection of subtle functional neuroadaptations associated with drug addiction.
